# Supplementary figures and images for: The gut microbiome of exudivorous marmosets in the wild and captivity
Source: Sci Rep. 2022 Mar 23;12:5049. doi: 10.1038/s41598-022-08797-7 (PMC8942988; doi:10.1038/s41598-022-08797-7)

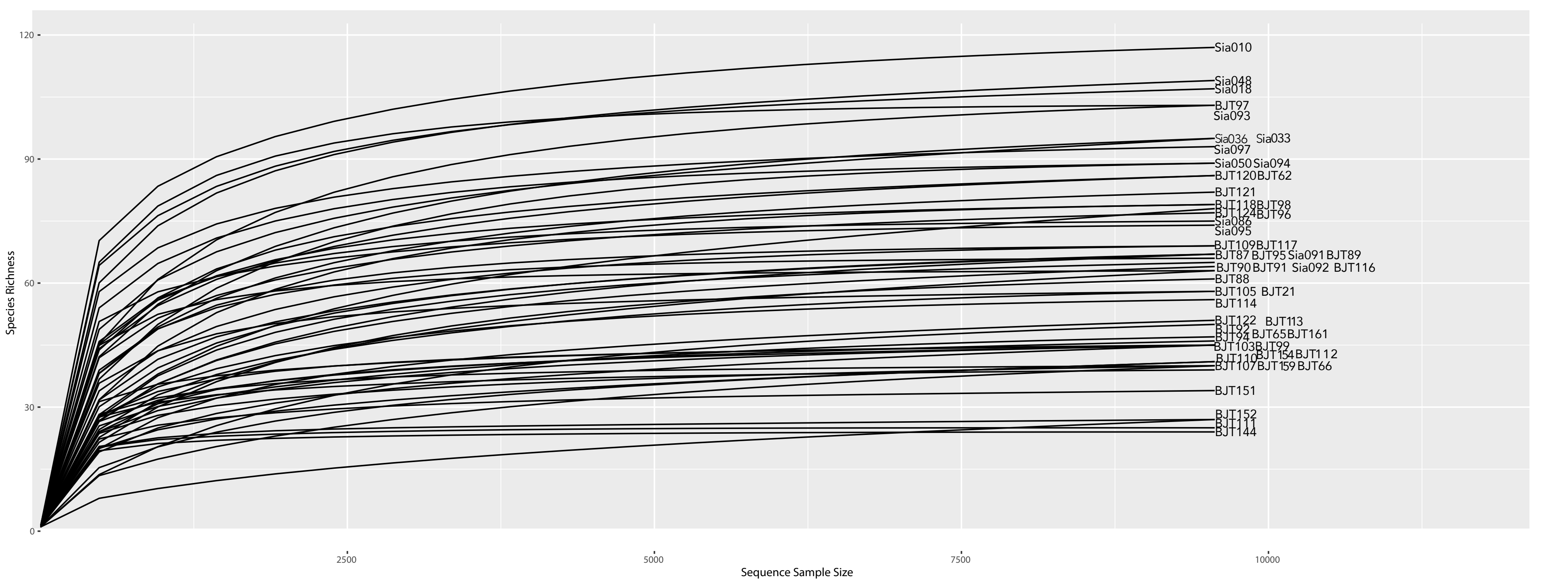

Supplement: Supplementary file 7 — Supplementary Figure S1. [file 41598_2022_8797_MOESM7_ESM.pdf]

A.

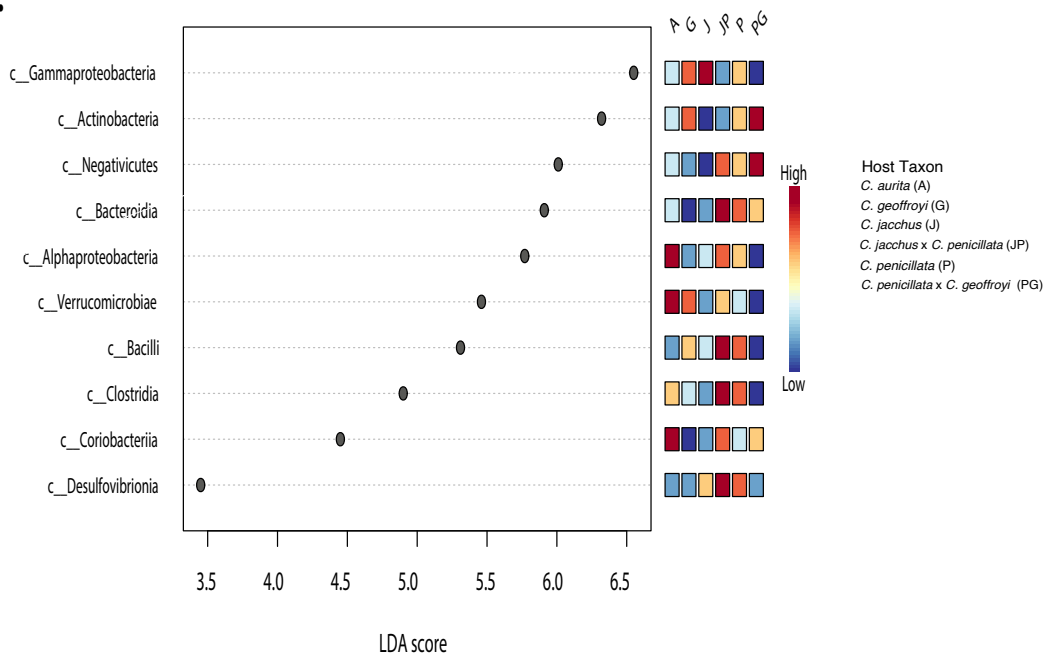

B.

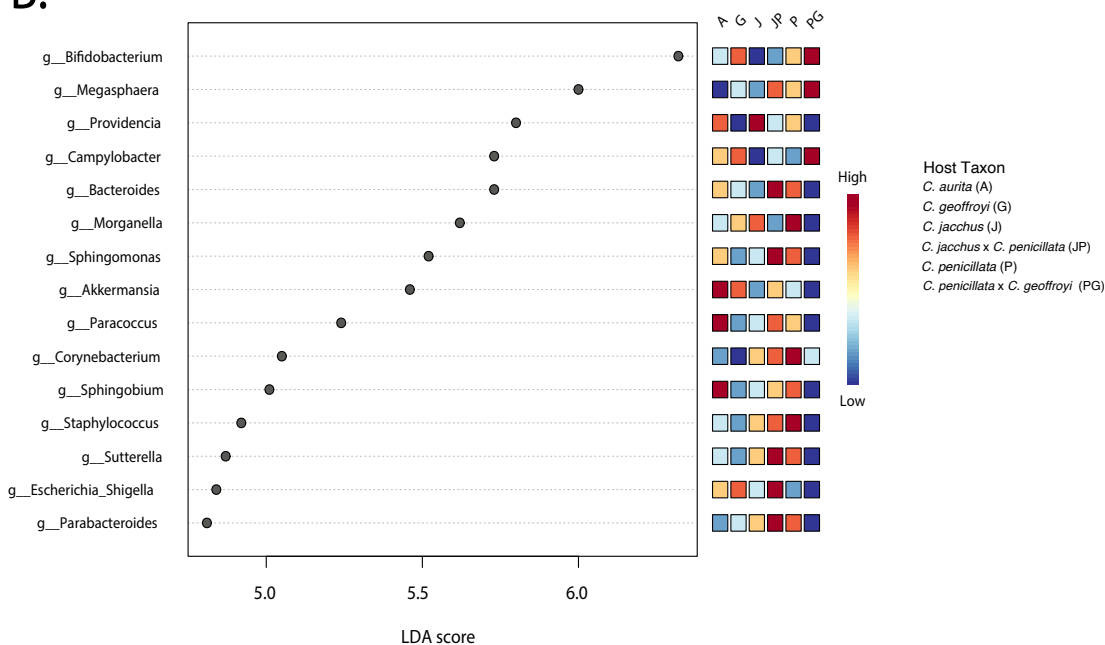

Supplement: Supplementary file 8 — Supplementary Figure S2. [file 41598_2022_8797_MOESM8_ESM.pdf]

A.

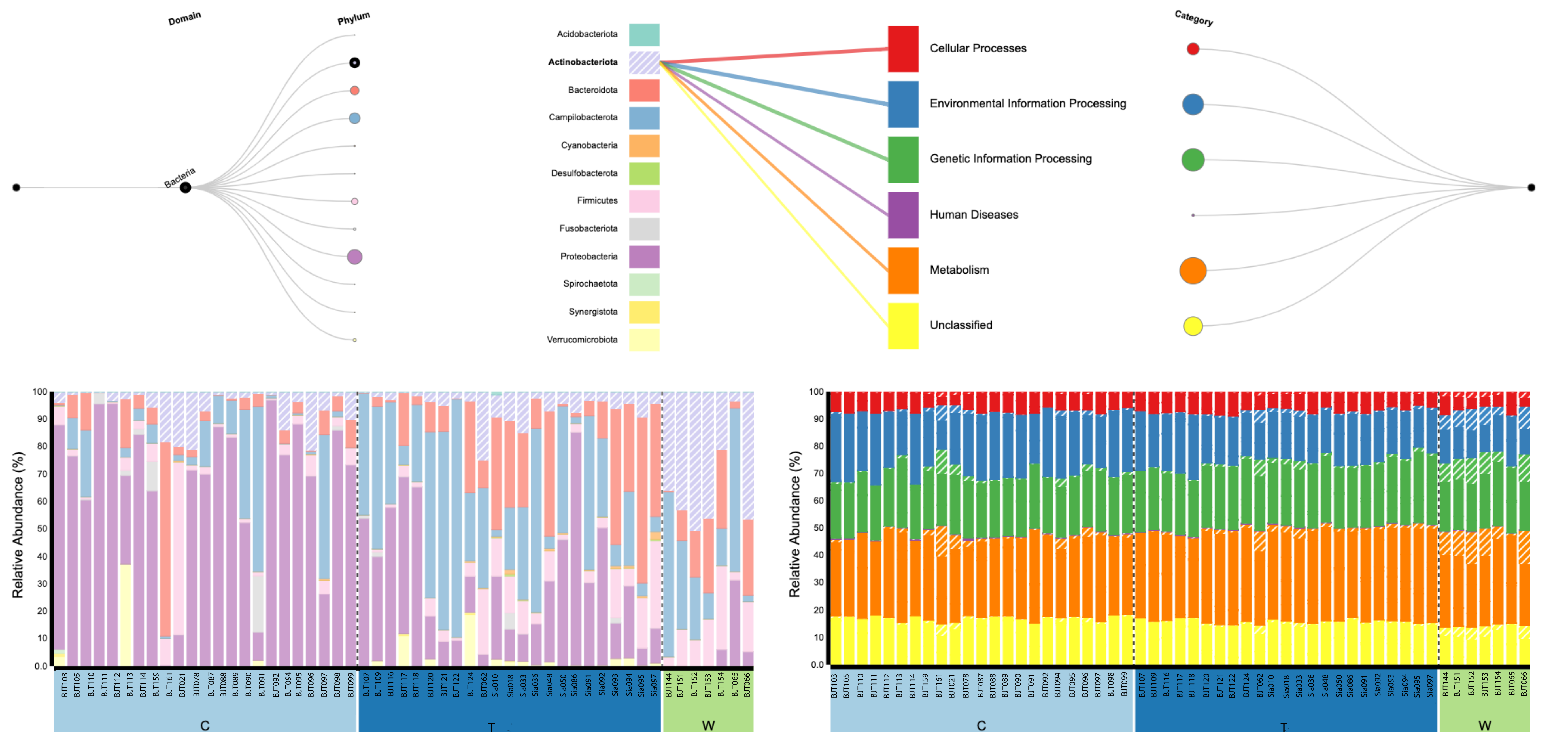

B.

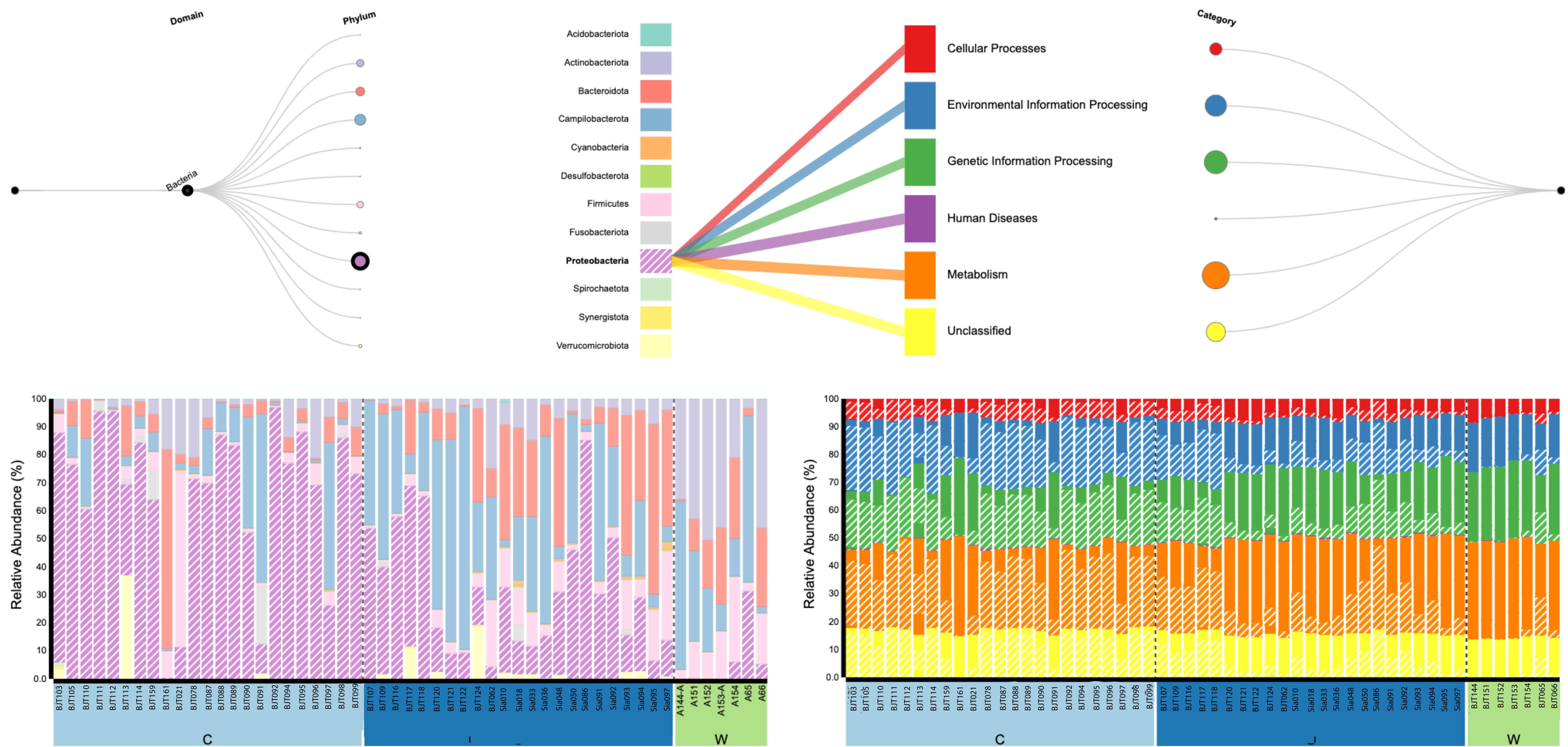

Supplement: Supplementary file 9 — Supplementary Figure S3. [file 41598_2022_8797_MOESM9_ESM.pdf]
