# Supplementary material for: The gut microbiome of exudivorous marmosets in the wild and captivity
Source: Sci Rep. 2022 Mar 23;12:5049. doi: 10.1038/s41598-022-08797-7 (PMC8942988; doi:10.1038/s41598-022-08797-7)
Supplement: Supplementary file 10 — Supplementary Figure S4. [file 41598_2022_8797_MOESM10_ESM.pdf]

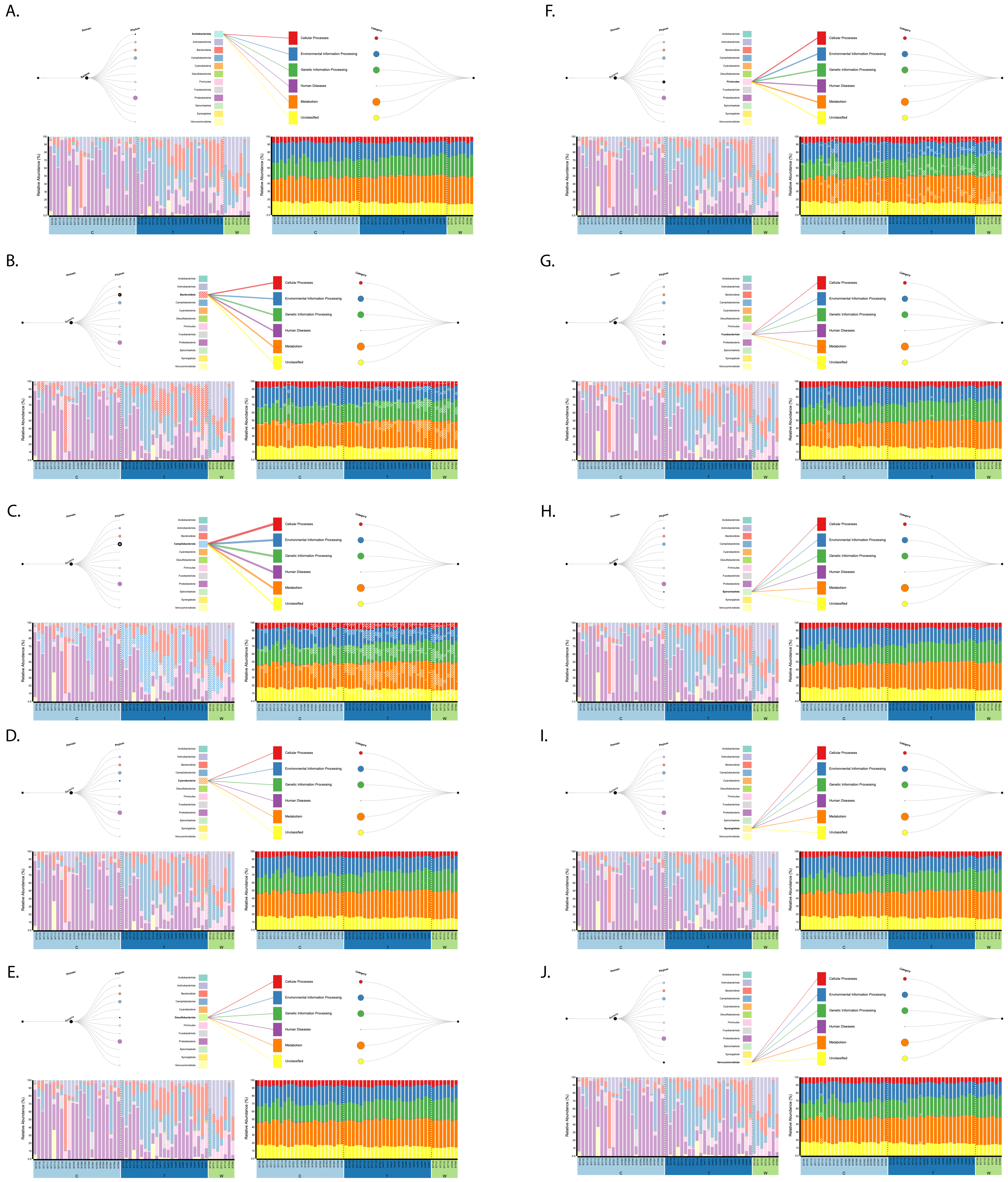
A.
B.
C.
D.
E.
F.
G.
H.
I.
J.
Domain
Phylum
Category
Bacteria
Acidobacteriota
Actinobacteriota
Bacteroidota
Campilobacterota
Cyanobacteria
Desulfobacterota
Firmicutes
Fusobacteriota
Proteobacteria
Spirochaetota
Synergistota
Verrucomicrobiota
Cellular Processes
Environmental Information Processing
Genetic Information Processing
Human Diseases
Metabolism
Unclassified
Relative Abundance (%)
C
T
W
